# Pathology of Jaundice

**Published:** 1875-01

**Authors:** 


Pathology of Jaundice.—Congestion of the liver is the cause to which jaundice is most frequently referred, when positive indications of other causes are wanting. M. Vulpian, a French writer, quoted by the *London Medical Record,* reviews the opinion of Broussais, who considered the most frequent cause to be an extension of inflammation from the mucous lining of the duodenum into the bile ducts. According to M. Vulpian, Virchow has confirmed this view, by detecting a plug of mucus in the common duct, near its orifice. The theory now is, that inflammation of the lining of the duct produces thickening and obstruction, to which the jaundice owes its causation.—*Pacific Medical and Surgical Journal.*

